# The exceptionally high rate of spontaneous mutations in the polymerase delta proofreading exonuclease-deficient *Saccharomyces cerevisiae *strain starved for adenine

**DOI:** 10.1186/1471-2156-5-34

**Published:** 2004-12-23

**Authors:** Alessandro Achilli, Nabil Matmati, Enrico Casalone, Giorgio Morpurgo, Angela Lucaccioni, Youri I Pavlov, Nora Babudri

**Affiliations:** 1 Dipartimento di Genetica e Microbiologia, Università di Pavia, Pavia, Italy; 2 Department of Molecular Pharmacology, St. Jude Children's Research Hospital, Memphis, TN 38105, USA; 3 Dipartimento di Biologia Animale e Genetica, Università di Firenze, Firenze, Italy; 4 Dipartimento di Biologia Cellulare e Molecolare, Università degli Studi di Perugia, Perugia, Italy; 5 Eppley Institute for Research in Cancer and Department of Biochemistry and Molecular Biology, University of Nebraska Medical Center, Omaha, NE 68198, USA

## Abstract

**Background:**

Mutagenesis induced in the yeast *Saccharomyces cerevisiae *by starvation for nutrilites is a well-documented phenomenon of an unknown mechanism. We have previously shown that the polymerase delta proofreading activity controls spontaneous mutagenesis in cells starved for histidine. To obtain further information, we compared the effect of adenine starvation on mutagenesis in wild-type cells and, in cells lacking the proofreading activity of polymerase delta (phenotype Exo^-^, mutation *pol3-01*).

**Results:**

Ade^+ ^revertants accumulated at a very high rate on adenine-free plates so that their frequency on day 16 after plating was 1.5 × 10^-4 ^for wild-type and 1.0 × 10^-2 ^for the Exo^- ^strain. In the Exo^- ^strain, all revertants arising under adenine starvation are suppressors of the original mutation, most possessed additional nutritional requirements, and 50% of them were temperature sensitive.

**Conclusions:**

Adenine starvation is highly mutagenic in yeast. The deficiency in the polymerase delta proofreading activity in strains with the *pol3-01 *mutation leads to a further 66-fold increase of the rate of mutations. Our data suggest that adenine starvation induces genome-wide hyper-mutagenesis in the Exo^- ^strain.

## Background

Mutagenesis in stationary-phase cells has attracted much attention recently. Historically, most studies on spontaneous mutation rates in bacteria and unicellular eukaryotes were conducted in exponentially growing cells, even though it was well known that bacteria and yeast, in their natural habitat, spend most of the time in the stationary phase [1,2]. There were relatively few papers about the accumulation of spontaneous mutations in non-dividing or poorly dividing cells [3] until the publication of the Cairns' paper "The Origin of Mutants" [4]. The paper showed that mutations can arise in a non-growing bacterial population and also suggested their adaptive nature. Since then, the parameters of spontaneous mutagenesis in unicellular organisms under conditions of limited growth have been examined in numerous studies [5,6]. Starvation for amino acids and bases has been used largely because of the relative ease of studying reversions of nutritional markers in both bacteria and yeast [4,7-9]. We previously reported that the replicative DNA polymerases δ and ε are involved in the control of mutability in non-dividing yeast cells. We have shown that strains with the mutational inactivation of proofreading exonucleases (*pol3-01 *and *pol2-4 *mutations, correspondingly) retained their mutator phenotypes, compared to each other and to the wild-type strains, upon histidine starvation [10]. This result was consistent with the earlier studies of the effect of the *cdc2-1 *mutation, allele of the *POL3 *[11].

We studied the effect of adenine starvation on the reversion of an auxotrophic strain carrying the *ade5-1 *allele. We show that adenine starvation induced a high level of reversion. The effect was further elevated 66 fold in the *pol3-01 *mutator strain, de3-01-CG. In addition to reversions, additional mutations throughout the genome were induced. The latter was suggested by the fact that most Ade^+ ^revertants were also auxotrophic for additional nutritional requirements and 50% of them were temperature-sensitive. These data suggested that reversions to prototrophy in the strain de3-01-CG, under adenine deprivation, are not adaptive, as was observed for histidine starvation [12]. We propose that adenine starvation of strains auxotrophic for this nutrient leads to perturbations of replication and/or DNA repair synthesis (for example, nucleotide pool deprivation or imbalances, which results in high rates of mutagenesis). The proofreading exonuclease activity of polymerase δ is an important factor that protects resting cells from this mutagenesis.

## Results

### Ade^+ ^revertants rates in the strains CG379-3-29(LR) and in de3-01-CG

**Figure 1:**
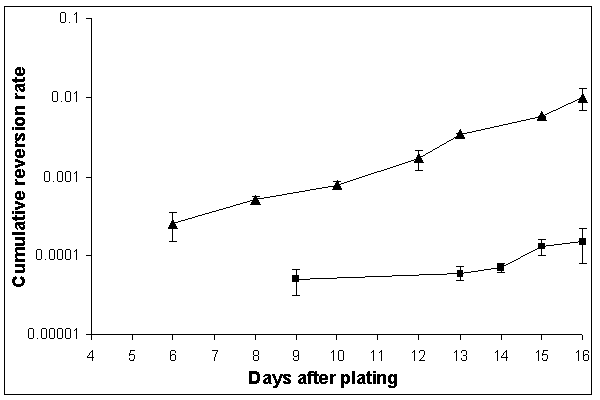
**Accumulation of Ade^+ ^revertants in the strains CG379-3-29 (LR) and de3-01-CG during starvation on SDNA-ade plates **The revertant rate for the strains CG379-3-29 (LR) (squares ■) and de3-01-CG (triangles ▲) is given as the total number of revertant colonies per plated cell. The mean values from 3 independent experiments are reported on a logarithmic scale (Y axis). Vertical bars represent the standard errors of the mean.

**Figure 2:**
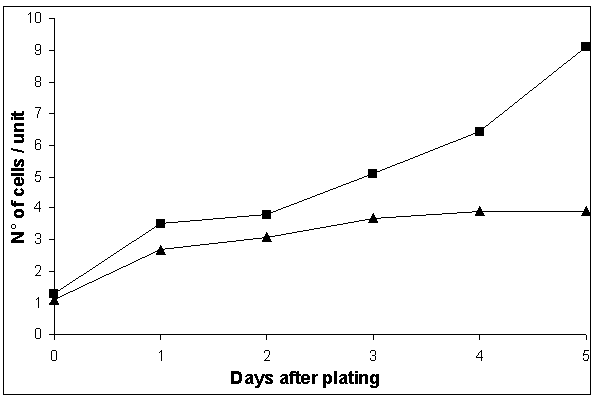
**Number of cells/unit on SDNA-ade in the strains CG379-3-29(LR) and de3-01-CG **The number of cells per unit was counted at the microscope (400×) in order to estimate the post-plating cellular divisions on SDNA-ade in the strains CG379-3-29 (LR) (squares ■) and de3-01-CG (triangles ▲).

**Figure 3:**
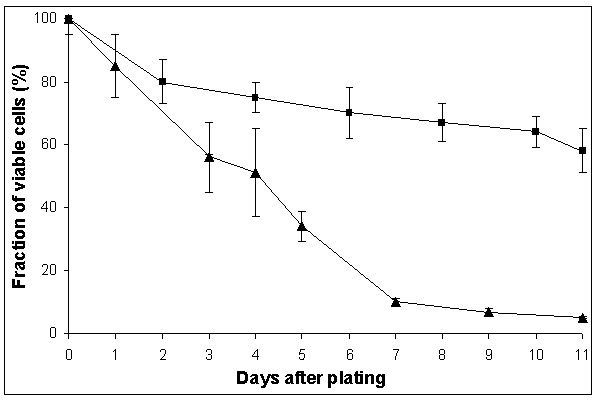
**Cell viability in the strains CG379-3-29 (LR) and de3-01-CG during starvation on adenine-free plates **The squares (■) represent values for CG379-3-29 (LR), while the triangles (▲) are values for de3-01-CG. The mean values from three independent experiments are reported. The vertical bars correspond to the standard errors of the mean.

First, we tested the effect of adenine starvation on reversion rates in a Hall's experiment [8], as we did in a previous study on histidine starvation [10,12]. The rate of accumulation of Ade^+ ^revertants on SD medium with limited amount of adenine was high: all colonies of the wild-type strain CG379-3-29(LR) on day 11, and its *pol3-01 *derivative with defect in proofreading by polymerase δ (strain de3-01-CG) on day 7, had one or more papillae, thus making it impossible to estimate the reversion rate. Therefore, we used the medium without any adenine (see "Methods"). High reversion rates were observed again, but an estimation of the mutation rate was now possible. Ade^+ ^revertants begun to appear on SDNA-ade plates on day 9 and on day 6 for the strains CG379-3-29(LR) and de3-01-CG, respectively, and continued to accumulate up to the end of the experiment (Figure 1). The cumulative reversion rate on day 16 was 1.5 × 10^-4 ^for CG379-3-29(LR) and 1.0 × 10^-2 ^for de3-01-CG. The presented reversion frequencies are the total number of Ade^+ ^revertants at the end of the experiment per cells plated (not per survived cells). There are several reasons why we did the calculations in this way. We do not know exactly when the mutational event occurred since the Ade^+ ^revertants growth rates were somewhat different (see below); we did not know the exact number of viable cells, due to ongoing residual divisions and cell death. In the latter respect, the strains CG379-3-29(LR) and de3-01-CG behaved very differently. The de3-01-CG cells stopped dividing on day 3 after plating and began to die. The strain CG379-3-29(LR) continued to multiply slowly and its survival was higher than survival of the de3-01-CG strain. These two parameters are documented in Figure 2 (dependence of number of cells on days of incubation) and in Figure 3 (cell viability versus time of incubation).

The differences in the growth rate among Ade^+ ^revertants made the estimation of the exact reversion rates in the logarithmic growth phase almost impossible. In one fluctuation test where 50 independent cultures of the de3-01-CG strain were analysed, we observed that the variability coefficient (σ × 100/X; where σ is the deviance and X is the mean) drastically dropped on day three. This suggests that the majority of Ade^+ ^revertants that arose during the logarithmic growth phase were able to give visible colonies by day 2. Therefore, we decided to calculate an approximate reversion rate on day 2 by the P_0 _method and obtained a reversion rate of 2.0 × 10^-7^, 4.5 orders of magnitude lower than the rate calculated on day 16 (see Figure 1). It was more difficult to determine the exact reversion rate in the logarithmic phase of growth for the CG379-3-29(LR) strain. Since the strain is not a spontaneous mutator, a very high number of cells had to be plated on SDNA-ade dishes to get reliable estimates. In a fluctuation experiment with 10^7 ^cells/dish, we did not observe any Ade^+ ^revertant colonies out of 50 independent cultures after two days of incubation at 28°C, implying a reversion rate lower than 2.0 × 10^-9^. Therefore, in this wild-type strain, starvation for adenine likely induced an accumulation of Ade^+ ^revertants at a level that is several orders of magnitude higher than in standard growth conditions.

### Molecular and growth characteristics of Ade^+ ^revertants

We compared the *ade5-1 *sequence from the CG379-3-29(LR) strain with the sequence of the *ADE5,7 *gene in SGD and found complete identity except for a C to A transversion at position 1158 (amino acid position 386). This transversion introduced the TAA ochre codon instead of the TAC tyrosine codon, generating a nonsense mutation.

DNA sequencing of the *ade5-1 *allele in 19 Ade^+ ^revertants of the de3-01-CG strain isolated on day 14 showed that they were all suppressors. Among 17 independent Ade^+ ^clones isolated on day 2 of a fluctuation experiment with the de3-01-CG (logarithmic cultures) strain, 6 were locus revertants and the rest were suppressors. The revertants obtained after starvation for adenine differed from each other in growth characteristics. Most of them gave visible colonies 6 to 8 days after plating on SDNA-ade. All of them had lower growth rates on both YEPD and SDNA+ade, than the parent de3-01-CG strain. The colonies were visible on YEPD at the stereomicroscope for even the best growing revertants 1 day later than colonies of the parent strain, de3-01-CG. These initial observations prompted a more detailed examination of the growth characteristics in different conditions.

### Are Ade^+ ^revertants adaptive?

The extremely high rate of Ade^+ ^revertants observed during prolonged incubation on selective medium could be the consequence of either "adaptive" or genome-wide mutagenesis in a strain with the *pol3-01 *mutation. To discriminate between the two possibilities, we tested the occurrence of temperature-sensitive (ts) and nutritional mutants among revertants and among Ade^- ^survivors. The results are described below.

#### a) ts mutants

The frequency of ts mutants in independent experiments was estimated using a random sample of de3-01-CG Ade^+ ^revertants, Ade^- ^survivors isolated on day 14 and Ade^+ ^revertants obtained from exponentially growing cells. The data are reported in Table 1. Fifty-one percent of Ade^+ ^revertants and thirty-nine percent of Ade^- ^survivors (i.e. non-mutated cells from aged and starved colonies) were ts mutants, whereas the number of ts mutants among exponentially growing cells was negligible. We also tested 100 Ade^+ ^revertants of the strain CG379-3-29(LR) starved for adenine and we did not find a single ts mutant.

**Table 1:** Ts mutant frequency among: Ade^+ ^revertants, Ade^- ^survivors and colonies from the log phase. The frequency of ts mutants in three independent experiments was estimated using a random sample of de3-01-CG Ade^+ ^revertants, Ade^- ^survivors isolated on day 14 and Ade^+ ^revertants obtained from exponentially growing cells. A hundred Ade^+ ^revertants of the strain CG379-3-29(LR) starved for adenine were used as controls. The percentage of ts mutants with respect to the number of tested colonies is reported in parentheses.

**Revertants/Survivors**	**No. of tested colonies**	**No. of ts mutants**
*Starvation condition day 14*		
de3-01-CG Ade^+^	489	249 (51%)
de3-01-CG Ade^-^	151	59 (39%)
CG379-3-29(LR) Ade^+^	100	0
*Log phase cells*		
de3-01-CG Ade^+^	1070	7^a^

^a ^The percentage has not been calculated since the ts mutants are likely to be a single clone.

### b) Nutritional mutants

To detect nutritional mutants we first used the replica plating technique ("Methods"), as we did for ts mutants. By this method, the fraction of nutritional mutants among de3-01-CG Ade^+ ^revertants was fifty-nine percent (20 out of 34 revertants tested); all were leaky nutritional mutants.

We did not systematically test the *ts *phenotype in these experiments, but we noticed that the two phenotypes, *ts *and the *nutritional requirement*, did not necessarily correlate.

**Figure 4:**
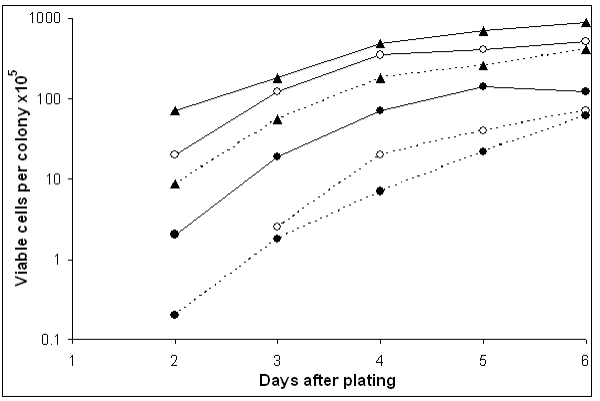
**Growth curves of the strains de3-01-CG and of two Ade^+ ^revertants on YEPD and SDNA+ade plates **The number of cells/colony (fitness) on SDNA+ade (dotted lines) and on YEPD (continuous lines) is plotted against days in culture. We compared the strain de3-01-CG (triangles ▲) with two Ade^+ ^revertants (strain 10, open circles ○; strain 15, closed circles ●).

To get a quantitative estimation of the severity of nutritional defects in Ade^+ ^revertants and Ade^- ^survivors, we compared their fitness ratios (number of cells per colony on SDNA+ade/number of cells per colony on YEPD; see "Methods") with the control strain, de3-01-CG. We presented the growth rate estimations for strain de3-01-CG and for two Ade^+ ^revertants in Figure 4. For all strains there was always less vigorous growth on SDNA+ade than on YEPD, however, the difference for Ade^+ ^revertants is much more distinct than the rate in the parent strain; the exponential growth phase ended on day 4. For this reason, we compared the fitness ratios for all strains on day 4. The data are reported in Tables 2 (Ade^+ ^revertants) and 3 (Ade^- ^survivors). Sixteen out of 17 Ade^+ ^revertants (94%) and 7 out of 12 Ade^- ^survivors had a two times lower fitness ratio than the control strain. It is important to note that all of the strains were respiratory competent, as determined by the 2,3,5-triphenyltetrazolium chloride (TTC) test. We concluded that Ade^+ ^revertants acquired one or more nutritional requirements.

**Table 2:** Number of cells/colony (fitness) of de3-01-CG Ade^+ ^revertants (1–17). We estimated the colonies' fitness as the number of cells per colony 30 on SDNA+ade as well as on YEPD after four days of incubation at 28°C. The strain de3-01-CG was used as control; see "Methods" for details.

**Strain/Revertant**	**No. of cells /colony (×10^7^) on:**		**Fitness ratio SDNA+ade/YEPD**
		
	**YEPD**	**SDNA+ade**	
de3-01-CG	4.380	1.700	0.388
1	1.700	-^a^	-^a^
2	0.070	-^a^	-^a^
3	-^b^	-^b^	-^b^
4	2.300	0.040	0.017
5	3.300	0.079	0.024
6	0.200	0.026	0.130
7	0.870	0.026	0.030
8	0.730	0.200	0.274
9	0.260	-^a^	-
10	3.900	0.200	0.051
11	1.700	0.300	0.176
12	1.700	0.210	0.124
13	2.600	0.100	0.038
14	1.600	0.200	0.125
15	0.700	0.080	0.114
16	0.500	0.080	0.160
17	1.700	0.210	0.124

^a ^Hardly visible at the stereomicroscope after 4 days of incubation at 28°C, diameter not estimable.

^b ^Not yet visible at the stereomicroscope after 4 days of incubation at 28°C. Visible colonies developed by day 8.

**Table 3:** Number of cells/colony (fitness) of Ade^- ^survivors isolated on day 14 from SDNA-ade dishes. see Table 2.

**Strain/Survivor**	**No. of cells/colony (×10^7^) on:**		**Fitness ratio SDNA+ade/YEPD**
		
	**YEPD**	**SDNA+ade**	
de3-01-CG	4.380	1.700	0.388
101	3.320	-^a^	-^a^
102	1.700	0.100	0.059
103	1.120	0.400	0.357
104	0.750	-^a^	-^a^
105	1.200	0.120	0.100
106	1.140	0.710	0.623
107	0.080	0.006	0.075
108	0.040	0.013	0.325
109	0.040	0.013	0.325
110	0.002	-^b^	-^b^
111	3.320	-^b^	-^b^
112	0.420	0.420	1.000

^a ^Hardly visible at the stereomicroscope after 4 days of incubation at 28°C, diameter not estimable.

^b ^Not yet visible at the stereomicroscope after 4 days of incubation at 28°C. Visible colonies developed by day eight.

With the replica-plating technique we estimated that the proportion of leaky nutritional mutants among revertants was 59% (see above). When the fitness ratios were determined, we concluded that 16 out of 17 Ade^+ ^revertants tested (94%) were nutritional mutants. We suggest that the differences in the estimates may be due to the inaccuracy of the replica plating technique. We did not find a single nutritional mutant among 12 log-phase revertants (data not shown).

In the next experiment, cells of the de3-01-CG strain were plated on SDNA without ade and containing four additional amino acids: val, ile, met, arg. We tested the proportion of clones requiring at least one of these amino acids by replica-plating and found that 10.9% of Ade^+ ^revertants and 7.9% of Ade^- ^survivors were auxotrophs for the selected four amino acids (Table 4; see "Methods" for more details).

**Table 4:** Frequency of auxotrophic mutants among de3-01-CG Ade^+ ^revertants and Ade^- ^survivors. Auxotrophic mutants were detected by replica plating de3-01-CG Ade^+ ^revertants and de3-01-CG Ade^- ^survivors on SDNA+ade and on SDNA+ade + (val, ile, met, arg). The percentage of auxotrophic mutants with respect to the number of tested colonies is reported in parentheses.

	**No. of replicated colonies**	**No. of auxotrophic mutants**
de3-01-CG Ade^+ ^revertants	375	41 (10.9%)
de3-01-CG Ade^- ^survivors	128	10 (7.8%)

### Does hypermutagenesis in the de3-01-CG strain under adenine starvation occur under starvation for other nutrilites?

To answer this question we evaluated the rates of reversion to prototrophy from histidine and tryptophan auxotrophy, respectively (the *his7-2*, frameshift allele; *trp1-289*, nonsense allele), by the same method that we used to evaluate the rates of accumulation of the Ade^+ ^revertants. We have previously shown that the reversion rate of the *his7-2 *allele in non-growing de3-01-CG cells was about 1.6 × 10^-7 ^on day 9 [10], therefore, we plated 1.0 × 10^6 ^cells/plate for our determinations. This density was optimized to avoid errors in rate estimation arising at higher densities due to cannibalism. The data reported in Table 5 suggest that histidine and tryptophan starvation was much less mutagenic than adenine starvation.

**Table 5:** Accumulation of de3-01-CG revertants under different selective conditions. The mean values of three experiments as well as the standard errors of the mean are reported; for each experiment, 15–20 dishes were done. The reversion rates are given as the total number of revertant colonies per plated cell.

**Selective Condition**	**Days after plating**					
	
	**6**	**8**	**9**	**10**	**12**	**16**
SDNA-trp	0.20 × 10^-6 ^± 1.00	0.26 × 10^-6 ^± 0.58	0.26 × 10^-6 ^± 0.58	0.73 × 10^-6 ^± 1.30	0.73 × 10^-6 ^± 1.30	0.73 × 10^-6 ^± 1.30
SDNA-ade^a^	2.50 × 10^-4 ^± 1.00	5.00 × 10^-4 ^± 0.60	-^b^	7.80 × 10^-4 ^± 0.90	1.70 × 10^-3 ^± 0.50	1.00 × 10^-2 ^± 0.30
SDNA- his	0.06 × 10^-6 ^± 0.58	0.12 × 10^-6 ^± 0.58	0.18 × 10^-6 ^± 1.17	0.18 × 10^-6 ^± 1.17	0.18 × 10^-6 ^± 1.17	0.18 × 10^-6 ^± 1.17

^a ^These values are from Figure 1.

^b ^Not determined.

## Discussion

In our previous studies we observed that the 3'→5' exonuclease activity of the polymerases δ and ε are both involved in correcting errors in yeast cells starved for histidine [10]. Here we investigated the effects of adenine starvation and the role of polymerase δ poofreading activity in resting cells starved for adenine.

At first we used the Hall's test [8], the same experimental approach as the previous paper [10], which allows us to detect revertants as papillae. As mentioned in the "Results" section, with the de3-01-CG strain, every colony had one or more papillae, making an estimation of the reversion rates by this method impossible. Therefore, we studied the rate of accumulation of revertants on SDNA-ade plates, a medium completely devoid of adenine. We have shown that the reversion rate rate in the wild-type strain CG379-3-29(LR) on day 16 was almost 5 orders of magnitude higher than the mutation rate estimated in a fluctuation test (where only the data of the first two days were considered). The high rate of mutations during adenine starvation was further elevated in the de3-01-CG strain, reaching 1% of the plated cells, which means an increase of 66 times with respect to the CG379-3-29(LR) strain. This implied that the proofreading activity of polymerase δ prevented a majority of mutations in resting cells, as in growing cells [13].

One possible explanation for the extremely high reversion frequency observed is that starvation of strains auxotrophic for adenine leads to perturbations of DNA replication and repair (for example, due to nucleotide pool deprivation or imbalances which are known to be mutagenic in yeast [14,15]). In the yeast *S. cerevisiae *the consequences of adenine starvation on mutagenesis were previously studied by Korogodin et al. [16]. The authors investigated the reversion rates of the *ade2-192 *allele (a missense mutation) in different strains and found that the lowest adenine concentration tested resulted in a 150-fold increase in locus revertants rate, while the suppressors rate was almost constant. When *ade2-192 *cells entered the stationary phase, their color shifted from white to red, a color which is due to a pigment that accumulates in *ade2 *mutants when the adenine biosynthetic pathway is in operation. Korogodin et al. [16] suggested, therefore, that the *ade2-192 *allele is derepressed under the condition of adenine deprivation and proposed that mutagenesis resulted from some process associated with transcription. Indeed, it is now well known that transcription-coupled mutagenesis occurs in yeast [17].

The data presented in this paper allow us to conclude that the high frequency of reversion to adenine prototrophy cannot be explained by transcription-coupled mutagenesis at the specific location of the *ade5-1 *allele in our experimental conditions. Instead, genome-wide mutagenesis occurred in the de3-01-CG strain under adenine deprivation. This is suggested by the high rate of ts and nutritional mutants among de3-01-CG Ade^+ ^revertants as well as Ade^- ^survivors. It is possible that genome-wide mutagensis occured in CG379-3-29(LR) as well but its lower mutability could have made difficult to detect ts and nutritional mutants even under adenine starvation. The observed mutation rates in the de3-01-CG strain were so high that we can characterize adenine deprivation as one of the most powerful mutagens for adenine auxotrophic strains. We can also conclude that under adenine starvation the 3'→5' exonuclease activity of polymerase δ prevented errors leading to the reversion of the *ade5-1 *strain to prototrophy along with the prevention of numerous genome-wide errors. Indeed, the rate of the *ade5-1 *reversion, under the conditions of adenine starvation was increased 66-fold in the *pol3-01 *strain. Apparently, the high rate of reversion was a sign of mutational catastrophe, since most of the revertants were ts or auxotrophs due to additonal mutations that occurred elsewhere in the genome.

High mutation rates, similar to what is described in the present paper, were reported by Bresler et al. [18]. The authors showed that bacterial cells grown on thymine-limited medium were often auxotrophs for more than one nutritional requirement, and that most mutants selected for other markers (such as streptomycin resistance) had additional mutations leading to auxotrophy. They observed, for example, that in an *E. coli *strain the percentage of streptomycin-resistant mutants with a nutritional requirement was 97.8%. This proportion is similar to the 94% of nutritional mutants obtained for de3-01-CG Ade^+ ^revertant clones in our study; however, Bresler et al. [18] performed experiments with replicating cells.

To the best of our knowledge, the high spontaneous frequency of ts mutations observed in the present work have never been reported. Hartwell [19], in a study of cells heavily treated with N-methyl-N'-nitro-N-nitrosoguanidine, found that 1% were ts mutants, which is fifty times lower than the proportion of ts mutants among the Ade^+ ^revertants reported here.

We can give a rough estimation of mutation rate per gene in the de3-01-CG auxotrophic strain under adenine starvation from the proportion of ts mutants (51%) among Ade^+ ^revertants. The ts phenotype is supposed to be the consequence of mutations in essential genes. According to Winzeler et al. [20], the number of essential genes in *S. cerevisiae *is about 1,000. If one Ade^+ ^revertant cell has a probability of 0.51 to be a ts mutant, then the probability for any one essential gene to mutate to a ts phenotype is 0.51/1000, i.e. 5.1 × 10^-4^. Harris and Pringle [21] observed that only a fraction of essential genes could be identified by ts mutations in *S. cerevisiae*. Since ts mutations are only a portion of total mutations, it is likely that each mutation rate in an essential gene was higher than the rate of ts mutations. This high mutation rate might be one of the reasons for a decrease in the viability of the *pol3-01 *strain under adenine starvation (see "Results"). We previously calculated that overall a 0.01 level of mutability leads to a drop of viability to 5% [22].

Finally, we wish to address a further important issue. In the present paper, we show that yeast haploid cells may sustain a very high genetic load even if their viability and fitness were reduced. Indeed, it is likely that the much lower survival of the mutator strain de3-01-CG, with respect to CG379-3-29 (LR) on adenine-free plates, was due to a high rate of mutations in the essential genes. One may wonder how some haploid cells can survive with such a high genetic load. One possibility is that under adenine starvation most mutations are base changes which could not have an appreciable lethal effect; actually, we found high rates of ts mutants which should be due to base changes. In natural conditions, yeast cells are diploids and, therefore, they could accumulate more variability, given that, according to Hartwell [19], 99% of ts mutations are recessive. Therefore, yeast diploids can afford much higher rates of mutagenesis [23-25]. The same holds true for other eukaryotic organisms. Pimpinelli et al. [26] showed that *Aspergillus nidulans *diploid conidia subjected to several cycles of 6-N-hydroxylaminopurine-induced mutagenesis (a base analog that induces only base substitutions) differ from each other for about ten lethals and, therefore, for a large number of mutations, perhaps several hundreds, without any viability reduction.

## Conclusions

In conclusion, we have demonstrated that: i) adenine starvation strongly induces reversion to Ade^+ ^phenotype in the wild-type strain; ii) a defect in the proofreading exonuclease activity of DNA polymerase δ due to the *pol3-01 *mutation leads to a further 66-fold elevation of the Ade^+ ^mutagenesis; and iii) under adenine starvation, the mutagenesis in the de3-01-CG strain is genome-wide, therefore, Ade^+ ^reversions in this strain are not adaptive.

## Methods

### Strains

The *S. cerevisiae *strains used were: CG379-3-29(LR) [*MATα ade5-1 leu2-3*,*112 *Δ *ura3 bik1::ura3 29 *(LR) *his7-2 trp1-289 CAN1 lys2-*Tn*5-13*]; and de3-01-CG [same as CG379-3-29(LR) but *pol3-01*] [27].

### Media

YEPD medium (1% Yeast Extract, 2% Pepton, 2% glucose) and the synthetic SD medium (6.7% Yeast Nitrogen Base, 2% glucose) were used throughout the work. In some experiments, SD was solidified with 1.5% Noble Agar (Sigma, SDNA) (see below). We named the SDNA medium containing all the nutrilites required by the strain SDNA+ade. When adenine was not included we named it SDNA-ade.

### Reversion rates and isolation of revertants

The accumulation of revertants under the starvation condition was investigated on SD with a limited amount of adenine (20 μg/l, SD.lim), as well as on SDNA-ade. On SD.lim we counted the number of colonies with Ade^+ ^papillae throughout the experiment [8]. On SDNA-ade plates, the number of Ade^+ ^revertants was evaluated as follows: 10,000 and 1,000 cells/plate were plated for the strains GC379-3-29(LR) and de3-01-CG, respectively, and incubated at 28°C; the revertant colonies were scored at the stereomicroscope (20×). For each experiment, 15–20 plates were set up. Noble agar was used to limit unwanted cellular divisions. The reversion rate is given as the total number of revertant colonies per plated cell. As explained in the "Results" section, we did not correct for the residual divisions or for the surviving fraction.

We estimated the total number of post-plating cellular divisions on SDNA-ade plates by comparing the number of cells immediately after plating and in the following days. The dishes were observed at the microscope (400×) and the number of cells per unit was counted. Here we considered a unit any single cell as well as more cells clustered together. For each experimental point, at least 200 units were counted and the mean values calculated.

To estimate the surviving fraction of cells and to characterise Ade^- ^survivors, we plated an adequate number of cells on SDNA-ade. We needed to rescue Ade^- ^survivors colonies from SDNA-ade dishes to determine viable cells and to isolate Ade^- ^clones for further characterisation. In order to do this, we have cut off two small pieces of agar in some dishes. This procedure created wells where we put adenine, which spread across plates without washing away the colonies. The procedure allows a correct estimation of surviving cells.

The reversion rate during the logarithmic growth phase was estimated by the fluctuation test using the P_0 _method [28,29].

To obtain Ade^+^revertants for further characterization we transferred Ade^+ ^colonies to YEPD medium. We were careful to pick up cells from the revertant colony by touching only the colony's surface with a needle at the stereomicroscope. Control experiments have shown that this procedure is reliable in our hands (data not shown). The phenotypic as well as the molecular analysis of Ade^+ ^revertants that arose in the logarithmic phase of growth was done on revertants isolated on day 2 in a fluctuation experiment. Only 1 colony per dish was picked up and isolated on YEPD. The analysis of Ade^+ ^revertants arisen in starved cells was done on a random sample of revertants isolated on day 14, since we were sure that the great majority of them derived from mutational events that occurred in cells starved for adenine (see "Results").

### Determination of growth of revertants on SDNA-ade

To determine the time of appearance of Ade^+ ^revertant colonies as well as their growth rate on SDNA-ade, we either plated 100 cells per dish or streaked suspensions (10^5 ^cells/0.1 ml), incubated at 28°C and scored at the stereomicroscope (20×).

### Detection of mutants with nutritional requirements

To detect leaky nutritional mutants among Ade^+ ^revertants and Ade^-^survivors, we estimated the colonies' fitness as the number of cells per colony [30] on SDNA+ade as well as on YEPD on fourth day after plating. Fifty cells of each strain were plated on Petri dishes containing 25 ml of YEPD and SDNA+ade, respectively. The plates were then incubated at 28°C and scored at the stereomicroscope for the appearance of colonies. Most colonies appeared by 4 days (see "Results") but the plates were incubated for longer to score slowly growing colonies. The diameters of 50 randomly chosen colonies were measured, and the mean value estimated. The mean value was used to calculate the approximate colony volumes, assuming their hemispheric shape as was done previously by Wloch et al. [30]. To obtain the number of cells per colony, we divided the colony volume by 1.1 × 10^-7 ^μl (by the volume of a haploid yeast cell) [31]. We then calculated the ratio of the revertant colonies' fitness on SDNA+ade to that on YEPD (SDNA+ade/YEPD) and compared it to that of the control strain. We arbitrarily considered those strains whose SDNA+ade/YEPD fitness ratio was at least twice lower than that of the strain de3-01-CG to be nutritional mutants. The comparison of SDNA+ade/YEPD ratios allowed us to detect nutritional mutants quantitatively but this approach was rather cumbersome. Alternatively, to screen more revertants, we used a qualitative test. The cells were point-inoculated by a needle on YEPD plates, which were then incubated for several days at 28°C. Thereafter, they were replica-plated on YEPD and SDNA+ade. After 2 days, the growth spots on both media were compared with those of the control strain. A revertant was considered a nutritional mutant when its SDNA+ade/YEPD growth ratio was reduced drastically (judged from visual inspection) with respect to that of the control strain.

The detection of respiration-deficient strains (*petite*) was done by the 2,3,5-triphenyltetrazolium chloride (TTC) test as in Ogur et al. [32].

### Detection of nutritional mutants auxotrophic for valine, isoleucine, methione, arginine

Strain de3-01-CG cells were plated on SDNA-ade plus other randomly chosen aminoacids [SDNA-ade + (val, ile, met, arg)]. On the day 14, the Ade^+ ^colonies were marked and adenine was added in two wells in agar as described above. The plates were incubated for further 6 days to allow Ade^- ^survivors to form colonies. Then they were replica plated on SDNA+ade and on SDNA+ade + (val, ile, met, arg) to detect auxotrophic mutants.

### Detection of temperature-sensitive (ts) mutants

Ade^+ ^revertants as well as Ade^- ^survivors isolated on SDNA-ade on day 14 were tested for the presence of ts mutations. To obtain Ade^- ^survivors, Ade^+ ^colonies were marked and then adenine was added to two wells of SDNA-ade plates seeded with 1,000 cells/plate on day 14. Dishes were incubated for 6 days more and previously non-marked colonies were isolated on YEPD. Colonies were then tested for their Ade^- ^phenotype on SDNA-ade.

To detect ts mutants, cells from a fresh culture were point inoculated by a needle on YEPD plates, which were incubated for several days at 28°C. Colonies were then replica plated on two YEPD dishes: one of them was incubated at 28°C while the other one at 37°C for two days. The first replica was always incubated at 37°C. A strain was considered to be a ts mutant if in two days it was able to grow at 28°C but not at 37°C. The strains CG379-3-29(LR) and de3-01-CG were included as controls.

### Determination of the nucleotide sequence of the ADE5,7 locus in CG379-3-29(LR) and Ade^+ ^revertants strains

Genomic DNA from *S. cerevisiae *was purified using the NucleoSpin^® ^Tissue kit (Macherey-Nagel).

The *ADE5,7 *locus (from 490 bp upstream of the start codon to 473 bp downstream of the stop codon) was amplified from genomic DNA of the CG379-3-29(LR) strain using primers ADE5-F1 (5'-CAAAAGTAGAAGACCCCC-3') and ADE5-R1 (5'-CCATTCATCAATTACGG-3'). The PCR reaction was performed according to standard protocol, using the proofreading-proficient Pfx DNA polymerase (Invitrogen). The PCR produced a DNA fragment of 3383 bp, which was purified from an agarose gel by the High Pure PCR Product Purification Kit (Roche). The nucleotide sequence of both strands of the purified DNA fragment was determined by the Sanger method [33], with fourteen different synthetic primers, using the BigDye Terminator Cycle Sequencing Kit (Applied Biosystem).

The sequence of the amplified fragment from the CG379-3-29(LR) strain was compared with the sequence of the *ADE5,7 *gene in the *Saccharomyces *Genome Database [34] by the BLAST algorithm [35].

DNA fragments of the *ADE5,7 *alleles in Ade^+ ^revertant strains were obtained by PCR amplification, using purified genomic DNA as template and the primers ADE5-F4 (5'-CCG TAA ACA TAG GAA TCG-3') and ADE5-R4 (5'-TTG TAC GAG ATT GTT ACC-3'). PCR, performed as previously described, generated a 398 bp long DNA fragment, encompassing the *ade5,7 *mutation in the CG379-3-29 strain. The purified DNA products were sequenced using the same primers, ADE-F4 and/or ADE5-R4.

## Authors' contributions

AA performed molecular analysis of Ade^+ ^revertants and AA, AL, NB and GM studied mutagenesis during starvation. YIP constructed the pol3-01 strain, participated in the design of the study and writing the manuscript. EC performed sequencing of the ade5-1 allele and molecular analysis of Ade^+ ^revertants. NB, GM and YIP coordinated the study. AA, AL, EC participated in the design of the study.

All authors read and approved the final manuscript.
